# Health sciences students knowledge, attitude and practices with chronic kidney disease in Jimma University, Ethiopia: cross-sectional study

**DOI:** 10.1186/s13104-019-4426-6

**Published:** 2019-07-11

**Authors:** Amare Desalegn Wolide, Kabaye Kumela, Fantu Kerga, Serkadis Debalke, Meskerem Seboka, Birtukan Edilu, Fanta Gashe, Eshetu Mulisa Bobassa

**Affiliations:** 1 Facility of Medicine, Jimma University, Jimma, 378 Ethiopia; 2 Faculty of Health sciences, Jimma University, Jimma, 378 Ethiopia; 3 Institute of Public Health, Jimma University, Jimma, 378 Ethiopia

**Keywords:** Chronic kidney disease, Knowledge, Attitude, Practice, Jimma University

## Abstract

**Objective:**

The objective of this study was to assess knowledge, attitude and practices of undergraduate health sciences students toward chronic kidney disease at Jimma University.

**Results:**

The overall weighted knowledge, attitude and practices score of the students were 8.6042 (8.26, 8.95), 6.23 (5.93, 6.53) and 2.51 (2.35, 2.67). Many students knew the basic function, symptoms and risk factors of chronic kidney disease. However, the same number of students showed a lack of diagnosis knowledge. Generally, students showed a favorable attitude and practice toward chronic kidney disease. However, they had a poor habit of a hospital visit for routine kidney checkup because of the socio-economic factors. The current study concludes that, despite students showed a good level of knowledge, attitude, and practices toward chronic kidney disease poor knowledge of kidney diagnosis methods and poor practice of visit to biomedical clinics for regular kidney checkup observed.

**Electronic supplementary material:**

The online version of this article (10.1186/s13104-019-4426-6) contains supplementary material, which is available to authorized users.

## Introduction

Chronic kidney disease (CKD) is defined as kidney damage or glomerular filtration rate (GFR) < 60 ml/min/1.73 m^2^ for three or more months with implications for health [1]. It is an increasingly prevalent health problem worldwide that may lead to poor outcomes of end-stage renal disease (ESRD) and cardiovascular disease [2]. Its incidence and prevalence have increased exponentially in recent years in both developed and developing nations [3]. It is consuming a huge proportion of health care finances in developed countries while contributing significantly to morbidity, mortality, and decreased life expectancy in developing ones [4–6]. Unfortunately, CKD problem remains underestimated on the entire continent due to lack of epidemiological information from different countries [5, 6]. For example, in Ethiopia, no study found focusing on knowledge, attitude and practice toward CKD among health sciences student. Current health sciences students are the future medical caregivers that could play an important role in the management of chronic kidney disease (CKD) at primary, secondary, and tertiary levels of health care centers. In order to perform their functions well, it is a must that they have a good understanding of CKD. Thus, we formulated a research question to asses’ knowledge, attitude and practice of students toward CKD among health sciences students in Jimma University. The outcome of the study will identify the areas of knowledge gap the students have and it will assist the students better for adequate health-care delivery to renal patients.

## Main text

### Methods

#### Study area and design

The study was conducted among Health Sciences undergraduate students in Jimma University, Ethiopia. The study was conducted from February 2018 to April 2018. Jimma University is one of the old Public University in Ethiopia located in the Southwestern part of Ethiopia just 352 km away from the capital city Addis Ababa. The Jimma University college of health sciences runs both undergraduate and graduates programs. Currently, the college has 9 undergraduate programs with a total number of 3356 students. The student in the department of Medicine, Pharmacy, Dental Medicine, Nursing, Anesthesia, and Medical Laboratory from year two to the graduating year included in the study without any pre-conductions. However, students in the department of environmental health, midwifery, and health officer were excluded from the study purposely.

#### Sample size determination

The sample size was calculated with single population proportion formula (using Z^2^ × p × q/d^2^) assuming the *p* value 50%, 95% confidence interval (CI) and 5% margin of error. In addition, considering a 10% non-response rate, the total sample size was 422.

#### Survey instrument

We did a systematic literature review to make questioners regarding knowledge, attitude, and practice related to kidney disease (KD). The questionnaire content was reviewed by three experts in the area after we did item analysis, difficulty with the items and item discrimination. Internal consistency and reliability were examined using Cronbach’s alpha greater than 0.62. Thus, the final version of the survey consisted of 33 items written in English. Knowledge domain was designed to test students understanding of the different aspect of kidneys. It comprised thirteen items and each item was categorized into a four-point response scale (‘Yes’, ‘No’, ‘Do Not Know’ and ‘Unsure’). The attitude domain contained eleven questions and each question has five-point categorical response scale (“Strongly agree”, “Agree”, “Not sure”, “Disagree”, “Strongly disagree”) to test students belief. The practice domain consisted of seven items and each question measured on a four-point Likert-based scale (‘Very Unlikely’, ‘Unlikely’, ‘Likely’ and ‘Very Likely’).

#### Data analysis

Statistical Package for the Social Sciences (SPSS) software version 23 (SPSS Inc., Chicago, IL, USA) was used for data analyses. Continuous variables are reported as mean and standard deviation (SD). Whereas, categorical variables as crude counts and percentages. Crude associations were estimated using generalized linear models. For each KAP domains, we calculated a composite score. For-example, for knowledge domain (range: 0–13), we first scored response “yes” as correct (1), and response “No” (0), “Do Not Know” (0) and “Unsure” (0) as incorrect (0). For the attitude domain, we scored as “Strongly agree” (1), ‘Agree’ (1) or ‘Not sure’ (0), ‘Disagree’ (0), and ‘Strongly disagree’ (0), and for the practice domain, ‘Very unlikely’ (1), ‘Unlikely’ (1), ‘Likely’ (0), or ‘Very likely’ (0).

### Results

Between January and May 2018, we enrolled 422 adults’ health sciences students from the second year to graduating year at Jimma University, Institute of Health Sciences. The total number of health sciences student according to the registrar office of health sciences was 3356. From the total 422 study participants, only 395 were completed the questioner perfectly. The reaming 27 study participant’s data were not entered in the data analysis due to the incomplete information they hold. Regarding the Socio-demographic characteristics, 257 (65.1%) were male and 138 (34.9%) were female in sex. Majority of the study participants 164 (41.5%) were from Medicine background and the smallest study participants 20 (5.1%) were from Dental Medicine background. The mean age of the study participants was 21.97 (± 2.33) and monthly income was 617.67 (± 1153.64) Ethiopian Birr (Table 1).

**Table 1:** Weighted knowledge, attitude, and practice score from separate univariable linear regression models among health science students of Jimma University; N = 395 (2018)

Variable	Frequency (%)	Mean (± Sta.d)	Knowledge score B (95% CI)	Estimated mean knowledge score (95% CI)
Sex				
Male	257 (65.1%)	–	0.88 (0.356, 1.41)*	8.916 (8.53, 9.30)
Female	138 (34.9%)	–	r	8.217 (7.81, 8.77)
Age	–	21.97 (± 2.33)	–	
Monthly income	–	617 (± 1153.64)	–	
Field of study				
Nurse	83 (21%)	–	− 1.489 (− 2.69, − 0.279)*	8.68 (8.11, 8.27)
Pharmacy	74 (18.7%)	–	− 1.22 (− 2.44, 0.009)	8.48 (7.86, 9.11)
Medicine	164 (41.5%)	–	− 0.496 (− 1.65, 0.654)	9.44 (9.00, 9.86)
Laboratory	25 (6.3%)	–	− 3.25 (− 4.71, − 1.79)*	7.42 (6.37, 8.47)
Anesthesia	29 (7.3%)	–	− 1.44 (− 2.85, − 0.025)*	8.34 (7.40, 9.29)
Dental medicine	20 (5.1%)	–	r	9.24 (8.12, 10.36)
Year of study				
Second year	124 (31.4%)	–	− 0.896 (− 1.366, − 0.426)*	7.66 (7.13, 8.12)
Third year	96 (24.3%)	–	− 0.281 (− 0.604, 0.043)	8.63 (8.08, 9.17)
Fourth year	86 (21.8%)	–	− 0.313 (− 0.56, − 0.067) *	8.49 (7.87, 9.11)
Fifth year	54 (13.7%)	–	0.047 (− 0.166, 0.261)	9.36 (8.65, 10.1)
Sixth year	35 (8.9%)	–	r	8.86 (7.95, 9.77)
Overall knowledge score				8.6042 (8.26, 8.95)
Overall attitude score				6.23 (5.93, 6.53)
Over all practice score				2.51 (2.35, 2.67)

*B* unstandarsized coefficients, *r* reference group

* Significant association (p < 0.05)

#### Knowledge

The overall knowledge score was 8.6042 (95% CI 8.255, 8.954) out of thirteen knowledge questions. Male students had a higher mean score 8.916 (95% CI 8.53, 9.30) than Female 8.217 (95% CI 7.81, 8.77). Medicine 9.44 (95% CI 9.00, 9.86) and dental medicine 9.24 (95% CI 8.12, 10.36) students showed highest mean knowledge score over other students. On year basis, fifth-year students 9.36 (95% CI 8.65, 10.1) and sixth-year students 8.86 (95% CI 7.95, 9.77) had highest mean knowledge score than others (Table 1).

Table 2 is demonstrating the knowledge questions responded by students. More than half of the students participated in the survey were aware of the role of the kidney in the human body (Q1). In addition, majority of the students knew the early symptoms (Q2) and potential risk factors (Q3–Q8) of kidney diseases (KD). Over half of 227 (57.5%) the students had wrong information related CKD diagnosis methods (Q9). In addition, 243 (61.5%) of the students had no information about the estimated glomerular filtration rate (eGFR) is the best indicator to assess the severity and function kidney (Q10). The large number 322 (81.5%) of the students they knew that CKD can be preventable by life modification (Q11) and low number of students 179 (45.3%) knew that end stage CKD has a treatment option kidney transplantation (Q12).

**Table 2:** Knowledge on normal kidney function, etiology, diagnosis, and treatment stratified by sex among health science students of Jimma University; N = 395 (2018)

Knowledge domain survey items		Sex		Total n = 395 (100%)
		Male n = 257 (65.1%)	Female n = 138 (34.9%)	
Q1. Waste removal… Function of kidney?	0	23 (5.8%)	30 (7.6%)	53 (13.4%)
	1	234 (59.2%)	108 (27.3%)	*342 (86.6%)*
Q2. Abdominal pain, urination difficulty and vomiting can be considered as early stages of CKD?	0	69 (17.5%)	51 (12.9%)	120 (30.4%)
	1	188 (47.6%)	87 (22.0%)	*275 (69.6%)*
Q3. Do you think holding urine cause kidney disease?	0	58 (14.7%)	43 (10.9%)	101 (25.6%)
	1	199 (50.4%)	95 (24.1%)	*294 (74.4%)*
Q4. DM and HBP cause kidney disease?	0	26 (6.6%)	31 (7.8%)	57 (14.4)
	1	231 (58.5%)	107 (27.1%)	*338 (85.6%)*
Q5. Do you think Urinary tract problems can cause kidney disease?	0	59 (14.9%)	39 (9.9%)	98 (24.8%)
	1	198 (50.1%)	99 (25.1%)	*297 (75.2%)*
Q6. Drinking little water may cause kidneys problem?	0	61 (15.4%)	40 (10.1%)	101 (25.6%)
	1	196 (49.6%)	98 (24.8%)	*294 (74.4%)*
Q7. Much salt consumption cause kidneys problem?	0	72 (18.2%)	47 (11.9%)	119 (30.1%)
	1	185 (46.8%)	91 (23.0%)	*276 (69.9%)*
Q8. High protein diet cause kidney problem?	0	112 (28.4%)	85 (21.5%)	197 (49.9%)
	1	145 (36.7%)	53 (13.4%)	*198 (50.1%)*
Q9. Does it possible know CKD by observing urine color or smell?	0	108 (27.3%)	60 (15.2%)	168 (42.5%)
	1	149 (37.7%)	78 (19.7%)	*227 (57.5%)*
Q10. eGFR is best diagnosis marker for CKD patients?	0	169 (42.8%)	74 (18.7%)	*243 (61.5%)*
	1	88 (22.3%)	64 (16.2%)	152 (38.5%)
Q11. Is it possible to…kidney disease early via life modification?	0	39 (9.9%)	34 (8.6%)	73 (18.5%)
	1	218 (55.2%)	104 (26.3%)	*322 (81.5%)*
Q12. Do you think that end stage CKD has treatment?	0	140 (35.4%)	76 (19.2%)	*216 (54.7%)*
	1	117 (29.6%)	62 (15.7%)	179 (45.3%)
Q13. Do you know where to go if you want a kidney screening test?	0	66 (16.7%)	50 (12.7%)	116 (29.4%)
	1	191 (48.4%)	88 (22.3%)	*279 (70.6%)*

Italic values indicate the highest number of the student

0-Incorrect, 1-Correct (Yes)

#### Attitude

Overall students showed a favorable response for attitude questions. For example, the majority 365 (92.4%) of the students agree or strongly agree on the possibility of living with one kidney if the other is removed for a health-related issue (Q1). Over half, 250 (63.3%) of the students believed renal function screening test is costly (Q2). However, majority 277 (70.1%) students had positive belief to go to health facility for screening if the local hospitals arrange free service or if someone can cover the cost of the test (Q3). The large proportion 322 (81.5%) of the students showed interest to learn all about CKD (Q5), however, 291 (73.7%) and 271 (68.6%) of the students had a frequent concern on stigma and treatment cost (Q6 and Q7) if they are diagnosed with the disease. About religion and kidney transplantation, most 245 (62.0%) of the students believed that religion does not influence kidney translation (Q8). Furthermore, students gave their views on the current prevalence of CKD and work performance of the Ministry of Health in Ethiopia. Therefore, majority 258 (65.3%) of the students did not believe that CKD is a major health problem in Ethiopia (Q9) but, the attention given to the diseases by the Ministry of Health is also very low (Q10). Finally, 306 (77.5%) of the student told they don’t believe the myth believed by the society that being a CKD patient could lead to death from any cause (Table 3).

**Table 3:** Attitude on chronic kidney disease stratified by sex among health science students of Jimma University; N = 395 (2018)

Attitude domain survey items		Sex		Total n = 395 (100%)
		Male n = 257 (65.1%)	Female n = 138 (34.9%)	
Q1. A person can live with one kidney?	1	240 (60.8%)	125 (31.6%)	*365 (92.4%)*
	0	17 (4.3%)	13 (3.3%)	30 (7.6%)
Q2. Do you think…..kidney screening test is expensive?	1	162 (41.0%)	88 (22.3%)	*250 (63.3%)*
	0	95 (24.1%)	50 (12.7%)	145 (36.7%)
Q3. I like to make Kidney screening test immediately if someone pays the cost for me?	1	178 (45.1%)	99 (25.1%)	*277 (70.1%)*
	0	79 (20.0%)	39 (9.9%)	118 (29.9%)
Q4. Have you thought you may have…signs or symptoms?	1	110 (27.8%)	75 (19.0%)	185 (46.8%)
	0	147 (37.2%)	63 (15.9%)	*210 (53.2%)*
Q5. I like learning all about kidney problems?	1	213 (53.9%)	109 (27.6%)	*322 (81.5%)*
	0	44 (11.1%)	29 (7.3%)	73 (18.5%)
Q6. I will be stigmatized by friend and families if i have kidney problem?	1	187 (47.3%)	104 (26.3%)	*291 (73.7%)*
	0	70 (17.7%)	34 (8.6%)	104 (26.3%)
Q7. I will be worried about treatment cost related with kidney disease?	1	173 (43.8%)	98 (24.8%)	*271 (68.6%)*
	0	84 (21.3%)	40 (10.1%)	124 (31.4%)
Q8. My religion does not allow kidney transplantation?	1	99 (25.1%)	51 (12.9%)	150 (38.0%)
	0	158 (40.0%)	87 (22.0%)	*245 (62.0%)*
Q9. Do you think that kidney disease is a major problem in Ethiopia?	1	95 (24.1%)	42 (10.6%)	137 (34.7%)
	0	162 (41.0%)	96 (24.3%)	*258 (65.3%)*
Q10. Do you think the Ethiopian Ministry of Health gave adequate…. Its prevention?	1	78 (19.7%)	56 (14.2%)	134 (33.9%)
	0	179 (45.3%)	82 (20.8%)	*261 (66.1%)*
Q11. Having chronic kidney disease… from any cause?	1	52 (13.2%)	37 (9.4%)	89 (22.5%)
	0	205 (51.9%)	101 (25.6%)	*306 (77.5%)*

Italic values indicate the highest number of the student

1-Agree and strongly agree, 0-Unsure, disagree and strongly disagree

#### Practices

About students practices, 328 (83.09%) of them would prefer to visit biomedical clinics (Q3) for renal function test over other options such as home treatment (Q2) and traditional healers (Q1). Student information seeking behavior about CKD was high. The primary sources of information for the majority of the students were textbooks and internet (Q4, Q5) (Additional file 1: Table S1).

### Discussion

Overall, over half of the students responded the correct answer on kidney basic function, symptoms, risk factors and preventive possibilities of the disease despite gaps were observed in identifying laboratory diagnosis methods. The good knowledge of the students seen in this study might be students had strong biology background in high-school as well as in the first year anatomy and physiology course. However, students showed poor knowledge of those questions related to those laboratory diagnosis methods. This could be students were not thought very well in clinical and laboratory course. The responses of the students on the different part of attitude questions were reflected as helpful. Majority of the students wish to visit a hospital or any biomedical clinics for routine kidney function test but they couldn’t because they have no adequate income to pay for the test. Due to this, most of the student had no habit of regular kidney function test. In Ethiopia, access to a health care center is extremely difficult due to socio-economic factors. In parallel to this, many students expressed their worry for they found themselves as renal patients. They thought they might be stigmatized by friends. In addition, they also told they would be worried because of the treatment cost. Students had a truth because people’s awareness about the disease is poor as well as treatment cost for the disease is beyond the capacity of any middle to high-income Ethiopian. Being a patient of CKD in the society is considered as weak, incapable of work and dying soon from any cause. We also have seen a huge number of students are not aware of the current magnitude and severity of the diseases in Ethiopia. Virtually no published reports found on the incidence and prevalence of kidney disease in Ethiopia. So that students believed, CKD is a disease of rich and westerns. However, many children and adults passed away from a poor family in Ethiopia. Therefore, the diseases are now becoming very serious especially in developing countries in association with the increase of diabetes and hypertension incidence. Thus, the federal ministry of Health of Ethiopia should work hard to increase people’s awareness about the diseases and associated factors. Related with practices, the majority of the students preferred to visit biomedical clinics for renal treatment than staying at home or going for traditional healers. Students also showed good practices of searching CKD related issues from textbooks to advance their understanding. Finally, we believe that our study has an important message to strength, promote and advance the knowledge and practice of nephrology. Today’s health science students are tomorrow’s caregiver for patients. So having good knowledge in the area is helpful to manage complicated health issues like CKD. Furthermore, these results may also be externally generalizable across other health sciences college students in the region as well as in the country.

### Conclusion

The student showed adequate knowledge, attitude and practices toward CKD. The poor knowledge observed in the diagnosis methods and the unfavorable attitudes and practices of the student should be change with reading and learning about the subject matter in detail.

## Limitations

We understood that our study has some limitations. The first is the nature of the study design. In cross-sectional study, the causal inferences may not be drawn and associations can be influenced by confounding variables. The second limitation of the study may be study subject selection bias. We used random sampling methods where only voluntary subjects were participated. Finally, we could not able to compare our study with other studies in the region due to lack of information in the area.

## Additional file

**Additional file 1: Table S1.** Practice on chronic kidney disease stratified by sex among Health Science Students of Jimma University; N = 395 (2018).

## Data Availability

The datasets supporting the conclusions for this study are available from the corresponding author on reasonable request.

## Abbreviations

eGFR: estimated glomerular filtration rate
GFR: glomerular flitration rate
KAP: knowledge, attitude and Practice
CKD: chronic kidney disease
KD: kidney disease
Epi Info: epidemiological information
