# Supplementary material for: Health sciences students knowledge, attitude and practices with chronic kidney disease in Jimma University, Ethiopia: cross-sectional study
Source: BMC Res Notes. 2019 Jul 11;12:389. doi: 10.1186/s13104-019-4426-6 (PMC6624927; doi:10.1186/s13104-019-4426-6)
Supplement: Supplementary file 1 — Additional file 1: Table S1. Practice on chronic kidney disease stratified by sex among Health Science Students of Jimma University; N = 395 (2018). [file 13104_2019_4426_MOESM1_ESM.docx]

**Table S1: Practice on Chronic Kidney disease stratified by sex among Health Science Students of Jimma University; N = 395 (2018).**

| **Practice Domain Survey Items** | | **Sex** | | **Total**  **n=395(100%)** |
| --- | --- | --- | --- | --- |
|  |  | **Male**  **n=257(65.1%)** | **Female**  **n=138(34.9%)** |  |
| **Q1. How likely you seek care from traditional healer?** | 1 | 224(56.7%) | 119(30.1%) | **343(86.8%)** |
|  | 0 | 33(12.8%) | 19(4.8%) | 52(13.2%) |
| **Q2. How likely you treat yourself at home?** | 1 | 181(45.8%) | 97(24.6%) | **278(70.4%)** |
|  | 0 | 76(19.2%) | 41(10.4%) | 117(29.6%) |
| **Q3. How likely you seek care at a hospital or from health care professionals?** | 1 | 45(11.4%) | 22(5.6%) | 67(17%) |
|  | 0 | 212(53.7%) | 116(29.4%) | **328(83.0%)** |
| **Q4. How likely you get information from textbook about CKD?** | 1 | 81(20.5%) | 47(11.9%) | 128(32.4%) |
|  | 0 | 176(44.6%) | 91(23.0%) | **267(67.6%)** |
| **Q5. How likely you get information about CKD from Social media?** | 1 | 57(14.4%) | 34(8.6%) | 91(23.0%) |
|  | 0 | 200(50.6%) | 104(26.3%) | **304(77.0%)** |

**Key: 1-**Very Unlikely and Unlikely, 0-Very Likely and Likely
